# A Low Frequency Electromagnetic Sensor for Indirect Measurement of Glucose Concentration: *In Vitro* Experiments in Different Conductive Solutions

**DOI:** 10.3390/s100605346

**Published:** 2010-05-28

**Authors:** Andrea Tura, Stefano Sbrignadello, Domenico Cianciavicchia, Giovanni Pacini, Paolo Ravazzani

**Affiliations:** 1 ISIB-CNR, Corso Stati Uniti 4, 35127 Padua, Italy; E-Mails: sbrignadello@isib.cnr.it (S.S.); giovanni.pacini@isib.cnr.it (G.P.); paolo.ravazzani@polimi.it (P.R.); 2 Bellco S.r.l., Via Camurana 1, 41037, Mirandola (MO), Italy; E-Mail: domenico.cianciavicchia@bellco.net

**Keywords:** dielectric properties, conductivity, sodium-chloride solution, Ringer-lactate solution, non-invasive glucose monitoring, non-contact sensor

## Abstract

In recent years there has been considerable interest in the study of glucose-induced dielectric property variations of human tissues as a possible approach for non-invasive glycaemia monitoring. We have developed an electromagnetic sensor, and we tested *in vitro* its ability to estimate variations in glucose concentration of different solutions with similarities to blood (sodium chloride and Ringer-lactate solutions), differing though in the lack of any cellular components. The sensor was able to detect the effect of glucose variations over a wide range of concentrations (∼78–5,000 mg/dL), with a sensitivity of ∼0.22 mV/(mg/dL). Our proposed system may thus be useful in a new approach for non-invasive and non-contact glucose monitoring.

## 1. Introduction

In the last two decades several studies have been carried out to establish non-invasive techniques for evaluation of glycaemia, *i.e.*, techniques not requiring blood collection [1–4]. In recent years, the assessment of the possible glucose-induced variations of blood dielectric properties has been suggested as a new non-invasive approach to determine glycaemia [5–9]. In [5], impedance variations were detected in glucose-water samples with different glucose concentrations, and similar results were found in our previous study [6], where we investigated the impedance variations in some glucose-water and glucose-sodium chloride samples (and also in some glucose-blood samples). In the study reported in [7], it was shown that variations in blood glucose concentration determine significant changes in the impedance of a subject’s skin and underlying tissues, though according to the authors, this was not directly due to variations in glucose concentrations, but rather to biochemical reactions across the membrane of erythrocytes triggered by glucose changes. Based on the assessment of tissues and blood dielectric properties, some prototypes for non-invasive glucose monitoring were developed [7–9], but currently no such device is commercially available.

All the studies quoted above [5–9], both *in vitro* and *in vivo*, were based on the analysis of possible variations induced by glucose, directly or indirectly, in the dielectric properties of solutions (possibly similar to blood or blood itself), and of human tissues, such as skin, underlying tissues and blood vessels. The common factor of all these studies was the use of electrodes, though of different shapes and properties, which were in contact with the medium (solution or tissue) to be studied. Unfortunately, the use of electrodes has some limitations, and it is prone to problems, such as the electrode polarization phenomena, that may affect the reliability of the results if not adequately taken into account and compensated for [10,11]. Also, in a possible application on humans, as foreseen in the prototypes [7–9], the use of electric currents flowing through the skin, though at very low intensity, may cause some undesired effects, such as skin irritation, widely reported with the use of the GlucoWatch device [1,2], which was a non-invasive glucose monitoring device based on reverse iontophoresis that was on the market for some years but then withdrawn (it must be acknowledged, however, that in iontophoresis the properties of the electric currents, in terms of intensity and frequency, may be different from those used in the prototypes described in [7–9]).

In order to avoid the limitations and problems indicated above (especially the risk of electrode polarization), an interesting approach for the analysis of the glucose-induced dielectric property variations could be the use of electromagnetic sensors rather than electrodes, thus avoiding any direct contact with the medium to be investigated. In this study, we present a novel electromagnetic sensor that we have recently developed, and we analyze its capability for the assessment of variations of glucose concentration in different solutions, namely, sodium chloride and Ringer-lactate solutions. The interest in this type of solutions is due to their clinical use in humans, and to their similarities to some extent with blood, as clarified below.

## 2. Experimental Section

### 2.1. Preparation of Samples

For the preparation of the first set of samples, 0.9% sodium chloride solution (Baxter) and d-glucose (99.5%, Fluka) were used. First, 5 g of glucose were added to 100 mL of 0.9% sodium chloride to give a final concentration of 5,000 mg/dL. Then, the dielectric properties of the solution were measured using the electromagnetic measuring system described in the following section. The 100 mL sample was afterwards diluted, by eliminating half of the sample and adding the same quantity of pure 0.9% sodium chloride solution. After proper stirring the measurement with the electromagnetic system was repeated on the new sample. The process was iterated, and hence at the end seven samples were prepared and measured, with glucose concentration values of 5,000, 2,500, 1,250, 625, ∼312, ∼156, ∼78 mg/dL. The dielectric properties of a sample of pure 0.9% sodium chloride solution was also measured as a blank control.

A second set of samples was prepared starting again from 100 mL of 0.9% sodium chloride solution and adding 0.1 g of sodium chloride (J.T. Baker). Thus, we obtained a 1.0% sodium chloride solution. With the method previously described we then obtained and analyzed the samples at the different glucose concentration values indicated above. A third and fourth set of samples were similarly derived by further adding sodium chloride, so that sodium chloride 1.1% and 1.2% solutions were obtained.

Finally, the fifth set of samples was prepared by using Ringer-lactate solution (130 mmol/L sodium ion, 109 mmol/L chloride ion, 28 mmol/L lactate, 4 mmol/L potassium ion, 1.5 mmol/L calcium ion) (Bieffe Medital) and then obtaining the samples at the different glucose concentration values.

### 2.2. The Measuring System with the Electromagnetic Sensor

**Figure 1.**
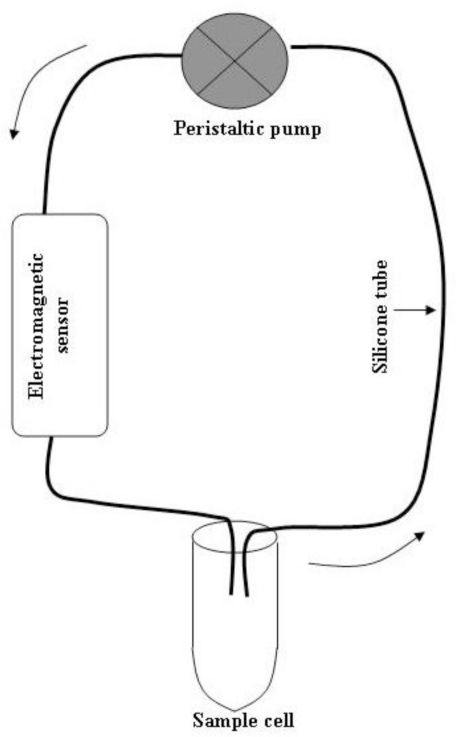
Block diagram of the measuring system.

**Figure 2.**
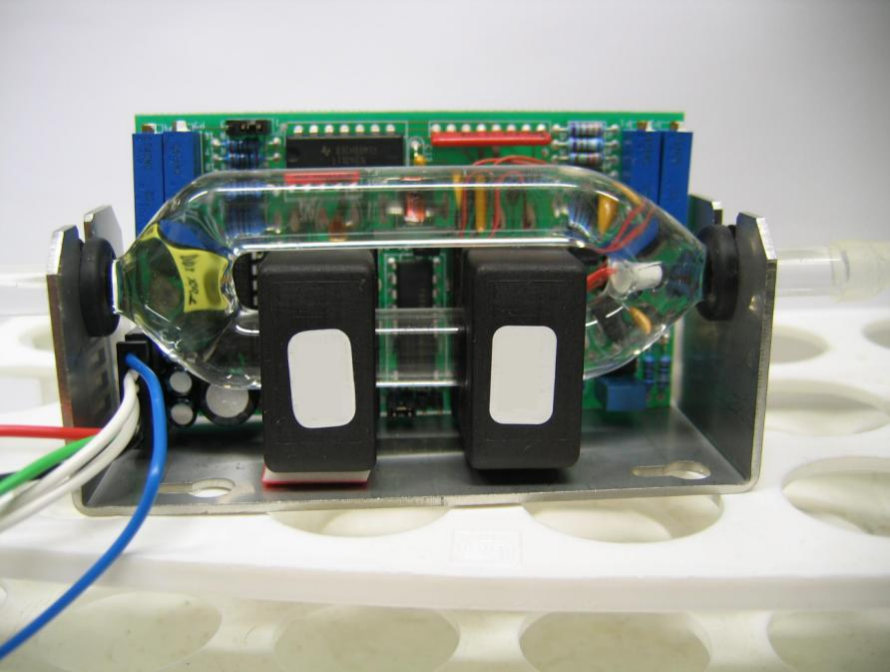
Photograph of the electromagnetic sensor.

The measuring system consisted of a peristaltic pump (Bellco), a glass cell containing the sample to be analyzed, a flexible silicone tube (diameter 8 mm, total length ∼200 cm) with both extremities within the glass cell (*i.e.*, immersed in the sample fluid), and an electromagnetic sensor inserted in the hydraulic circuit. The block diagram of the system is shown in Figure 1. The electromagnetic sensor has been recently developed at the Bellco R&D division, Mirandola (MO), Italy. A photograph of the sensor is shown in Figure 2.

The sensor is based on the physical principle of electromagnetic induction. Two induction coils are coupled through the medium under investigation (*i.e.*, the fluid flowing in the hydraulic circuit). The fluid represents the core linking the two inductors. The electromagnetic coupling of the two inductors is modified by variations in the dielectric parameters of the fluid. In fact, when the primary coil is excited from an AC source, a magnetic field in the primary coil induces an electric field around the primary and the secondary coils. This electric field induces an electric current in the fluid ring (the sensor has in fact two branches: see Figure 2). This current induces a magnetic flux in the secondary coil, and the magnetic flux induces an electromotive force in the secondary coil itself. The current in the fluid can be estimated by measuring the current in the secondary coil: *i_s_* (current at secondary coil) is equal to *i_f_*/*n_s_* (*i_f_* being the current in the fluid, and *n_s_* the number of secondary coil turns). Since the magnitude of the current in the fluid is proportional to the admittance of the fluid ring, the conductivity of the fluid can be derived. In theory, the permittivity of the fluid could also be measured with this method, but in practice such a kind of sensor is more adequate for the measurement of the conductivity [12].

In our sensor, the two inductors are realized with small size components (30 × 30 × 16 mm). We used commercial components, such as the Philips 4330-030-3752 (other components can be used alternatively). The primary inductor works in a push-pull configuration. It is driven by a regulating pulse width modulator (Intersil CA3524). This component was set to have the inductors working at 40 kHz. The electromagnetic sensor needs two power supply lines, at +15 V and −15 V. They were provided through a stabilized power supply generator with double output (E.S. Roland). The output of the electromagnetic sensor that we considered in this study was a DC electric current (from 0 to ∼25 mA). This current was directed over a 500 Ω (1/4 W, 1%) resistance, and hence we obtained a DC output voltage from 0 to ∼12.5 V. Thus, we simply analyzed the conductivity values of the studied samples. The relationship between the output voltage and the sample conductivity was given in all the investigated conductivity range by the equation 1 V = 2 mS/cm. This relationship was obtained by calibrating the sensor with known conductivity standards, considering that the k-factor of the sensor (*i.e.*, the volume of the fluid ring) is equal to 8 cm^3^. The peristaltic pump required a power supply at +15 V (or less, depending on the flow velocity that was needed). This was provided by a single output stabilized power supply generator (Stab Italy).

### 2.3. The Measurement Procedure

As regards the first set of samples (0.9% sodium chloride solution), we first analyzed the 5,000 mg/dL glucose sample. The peristaltic pump was initially powered at 10 V, thus giving a fluid velocity of around 20 cm/s. We waited for some seconds to allow filling of the hydraulic circuit with the sample fluid, and we verified that no air bubbles were present. In the case that some bubbles appeared, we eliminated them by gently shaking the silicone tube and/or the electromagnetic sensor manually until their complete disappearance. Through a digital multimeter (Wavetek) we verified the output voltage of the sensor, and saved the value in an electronic worksheet. To assess possible effects on the estimation of glucose concentration due to the flow velocity, we then increased the pump power to 15 V (the flow velocity in the circuit changed almost linearly with the pump power), and repeated the measurement. Furthermore, we then decreased velocity to 5 V and 0 V, thus also getting a measurement under static conditions (no flow). For each measurement, the temperature of the sample fluid was checked through a thermometer for fluids, with ±0.3 °C accuracy (Hanna Instruments). For all the measurements at room temperature (also for the other sample sets), the sample temperature was in the range of 22–23 °C.

After the four measurements at the different flow velocities, the circuit was emptied, and then filled with the blank solution (0.9% sodium chloride solution with no glucose); we circulated the solution for around one minute, and then emptied the circuit again. Then, we put in the cell the sample at the immediately lower concentration (2,500 mg/dL in this case), and repeated the whole procedure. We continued with all the samples, until the ∼78 mg/dL glucose sample, and finally we also performed the measurements on the blank sample. All the described procedures were performed for the second, third, fourth and fifth sets of samples. Of course, the washing operations of the circuit after each measure was performed with the specific blank of each sample set.

For two samples in each sample set, we also performed some measurements to assess the possible effect of temperature variations on glucose concentration assessment. We chose one high glucose level sample (2,500 mg/dL), and a low level one (∼156 mg/dL). By means of a heating plate (Velp Scientifica) we progressively heated the sample, and performed the measurement at around 30 °C, 37.5 °C (of special interest being the human body temperature), and 45 °C.

Of note, every measurement (*i.e.*, for every sample in each set, at the different velocities and, in some cases, different temperatures) was repeated twice, on separate days, starting all the experimental procedures from the beginning (*i.e.*, preparation of samples). Furthermore, as regards the 0.9% sodium chloride and ∼78 mg/dL glucose sample, we performed the measurement 10 times (pump powered at 10 V, room temperature), to get an indication of the repeatability (precision) of our approach (all measurements were performed by the same operator).

The conductivity of some of the studied samples was also measured with a commercial conductivity meter (Amel Instruments, mod. 160): first, we measured the blank sample of 0.9%, 1.0%, 1.1% and 1.2%, sodium chloride and of Ringer-lactate. Then, we measured the series of samples of 0.9% sodium chloride plus glucose at the different concentrations (from ∼78 to 5,000 mg/dL). Thus, a total of 12 samples were analyzed. Of course, the measurements with this meter were obtained by direct contact of the sample with the meter’s probe, which was in fact immersed directly into the sample cell filled with the fluid sample of interest. All the measurements were performed twice, at room temperature.

## 3. Results

**Figure 3.**
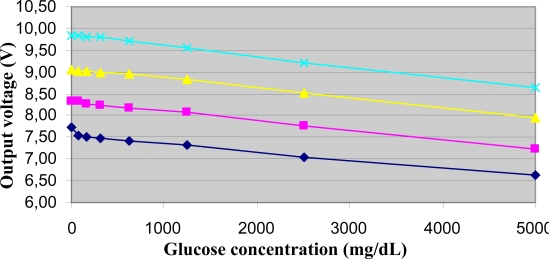
Output voltage of the electromagnetic sensor in relation to the glucose concentration of samples in the 0.9% sodium chloride set (rhombus), 1.0% set (square), 1.1% set (triangle), 1.2% set (x symbol), at room temperature and the pump powered at 10 V.

The output voltage of the electromagnetic sensor in relation to the glucose concentration of the samples in the sodium chloride 0.9%, 1%, 1.1% and 1.2% sets is reported in Figure 3 (for each sample, the average value between the related couple of measures is shown; this applies also to the other results presented).

It can be appreciated that in each set of samples the output voltage does in fact depend on the glucose concentration of the sample over the whole studied range of glucose concentration values. It can also be noticed that the output decreases almost linearly with the increasing glucose values. In fact, for the 0.9% sodium chloride set, regression analysis provided a high R^2^ value (R^2^ = 0.97, P < 0.0001), and similar results were found for the other three sets (not reported for brevity).

We also computed a sort of sensitivity index for the glucose variations, equal to S = ΔV/ΔG, where ΔV is the total difference in the output voltage, and ΔG is the total variation in the glucose range. In the 0.9% sodium chloride set, ΔV was equal to 1.11 V, whereas ΔG was equal to 5,000 mg/dL for any sample set; thus, S = 0.22 mV/(mg/dL). Of note, due to the almost linearity of the output-glucose curve, S also had very similar values when computed in smaller intervals of the glucose range: for instance, if we consider the glucose interval ∼78–625 mg/dL, the calculation of S provided 0.21 mV/(mg/dL). Similar results (not reported) were found for the other three sample sets.

**Figure 4.**
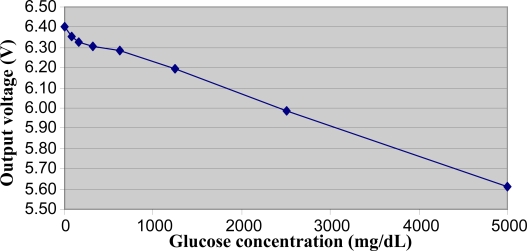
Output voltage of the electromagnetic sensor in relation to the glucose concentration of samples in the Ringer-lactate set, at room temperature and pump powered at 10 V.

The relationship between the output voltage of the electromagnetic sensor and the glucose concentration in the samples of the Ringer-lactate set is illustrated in Figure 4. Again, we found a clear decrease in the output for increasing glucose values, though the relationship in this case appeared to be non-linear, at least in the first part of the glucose range.

**Figure 5.**
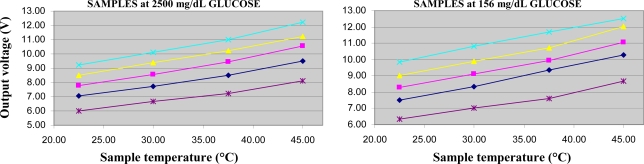
Output voltage of the electromagnetic sensor in relation to the sample temperature (room temperature, 30 °C, 37.5 °C, 45 °C) in the Ringer-lactate set (star), in the sodium chloride 0.9% set (rhombus), 1.0% set (square), 1.1% set (triangle), 1.2% set (x symbol), for fixed glucose concentration of 2,500 mg/dL (left) and ∼156 mg/dL (right), with pump powered at 10 V.

As regards the effect of temperature variations on the output voltage, as expected an increase of the output (*i.e.*, higher conductivity) was found for increasing temperature values (Figure 5). This was found both on the high glucose sample (2,500 mg/dL) and the low value one (∼156 mg/dL), in any sample set. The measured variations were in the range 1.4–1.6%/°C. These results appear consistent with what reported in the literature for temperature-induced conductivity variations of sodium chloride solutions [13].

The difference in the output of the electromagnetic sensor due to the flow velocities (from no motion to around 30 cm/s) was found to be small in all the sample sets, at any glucose concentration. In fact, the standard deviation of the four measures at the different velocity conditions was only 17 mV on average over all the samples analyzed in this study.

To derive an indication of the minimal glucose concentration difference that could be measured with our approach, we referred to the repeatability experiment based on the 10 measures performed on the 0.9% sodium chloride and ∼78 mg/dL glucose sample: considering three times the standard deviation of these measures, and given the S value reported above, we got a value of ∼30 mg/dL. This is not a negligible value; however, it includes not only the imprecision of the sensor, but also the possible uncertainties in some steps of the experimental procedure (in particular, imprecision in the dilution phase).

As regards the measurements obtained with the commercial conductivity meter, we found the results to be in very good agreement with those obtained with the electromagnetic sensor. The average conductivity value over the 12 measured samples was 16.04 mS/cm (standard deviation, SD = 2.10); the corresponding average value found with the electromagnetic sensor was 15.40 mS/cm (SD = 1.94). Regression line was Y = 0.91X + 1.03, R^2^ = 0.99, P < 0.0001. A Bland-Altman plot (not shown) showed no points outside the limits of agreement.

## 4. Discussion

We have presented a novel electromagnetic sensor, and we analyzed its ability in an *in vitro* context to estimate variations of glucose concentration in solutions that have some similarities with blood, *i.e.*, sodium chloride and Ringer-lactate solutions. The 0.9% sodium chloride solution was considered of interest as it has an osmolarity similar to that of plasma (300 mOsm/L *versus* ∼290 mOsm/L), and conductivity similar to that of blood. This solution is in fact used in intravenous therapy in subjects under acute dehydration or not able to assume fluids orally. The interest for the study of the Ringer-lactate solution was again due to some similarities with human fluids: it is isotonic with blood, and it has a chemical composition similar to that of interstitial fluids. Its use in humans is mainly for parental therapy of fluid loss, due to injuries, burns, or surgery; it is also used to stimulate urination in subjects with renal diseases and when some slight or moderate acidosis states must be corrected.

This study can be considered the first step to verify whether our electromagnetic sensor might be adequate as the basis for an approach for non-invasive monitoring of glycaemia, within the framework of the approaches based on the measurement of the dielectric properties of human tissues. In the recent years, some prototypes for non-invasive glucose monitoring have been developed [7–9] based on the estimation of tissues and blood dielectric properties, but currently no device is available for purchase. More precisely, the Pendra device from Pendragon Medical [7,14] also received the CE approval (thus it was ready for commercialization), but its diffusion in the market was prevented by some concerns about its actual performance [15]; other prototypes [8–9], though already under testing on humans, according to the information available at the company’s web site (www.solianis.com), do not seem to have acquired all the regulatory approvals yet. These prototypes, though very promising, are based on the use of electrodes in contact with the skin, which may potentially cause some problems. Apart for some possible side effects (such as skin irritation), the reliability of the measurements may be affected by phenomena due to the contact of the electrodes with the skin, in particular electrode polarization [10,11]. It is true that some suitable tricks could prevent or limit these problems: some black platinum electrodes can be used [16], or measurements can be performed at higher frequencies (above some hundreds of kHz). However, though the choice of relatively high working frequencies may solve the electrode polarization problems, some further problems or drawbacks (such as the possible skin irritation) may remain. The use of an approach based on electromagnetic fields generated by inductors not requiring contact with the medium to be studied would solve any possible problem of the electrode-based approach. That motivated us to develop and analyze the electromagnetic sensor presented in this study.

In this preliminary *in vitro* analysis, we studied different solutions with glucose concentration values varying from 5,000 to ∼78 mg/dL. These glucose values represented a range going from very high values, but nonetheless sometimes observed in humans (values above 1,800 mg/dL are common in a diabetic coma), to values typical of subjects with normal glucose tolerance [17]. The interest for the study of samples at different concentration of sodium chloride (from pure saline solution used for intravenous infusions (0.9%) up to 1.2%) was due to the fact that we also aimed to verify whether the performances of the electromagnetic sensor in the assessment of glucose variations in the solutions could be influenced by the degree of conductivity of the blank solution. Our results showed that the electromagnetic sensor was able to detect variations in glucose concentration in all the investigated wide glucose range, and at the different basal conductivity values of the four sodium chloride sample sets. Also, the relationship between the sensor output and the glucose concentration was essentially linear for all the sample sets.

With the study of the Ringer-lactate solution samples, we verified whether a chemical composition again typical of human fluids, but more complex than that of the sodium chloride solutions, may affect the electromagnetic sensor performances. We found that the electromagnetic sensor was again also able to detect variations in glucose concentration in the Ringer-lactate samples, though the relationship between the sensor output and the glucose concentration was not linear in the first portion of the investigated glucose range. At this stage we do not have a satisfactory explanation for this result, but one reason that may partially justify this finding is the possible uncertainty in the glucose concentration values of the samples obtained after several dilutions.

From our experiments we can conclude that variations in glucose concentration directly affect the conductivity of a solution, independently from some phenomena that in blood may be triggered by glucose variations, such as the hyperglycaemia-hyponatremia phenomenon [18] that may cause dielectric property changes due to sodium ion migration across the membranes of the erythrocytes. By studying solutions without erythrocytes or any cellular component we found that an increase in glucose concentration directly determines a decrease in the conductivity of the solution. From a qualitative point of view, the fact that glucose does not dissociate into ions may at least explain why it does not determine the opposite behavior (*i.e.*, conductivity increase), which in fact we have never observed.

The first and, to our knowledge, only studies where an electromagnetic approach was proposed for possible non-invasive measurement of glucose concentration values were those reported in [19,20]. Like in our study, the authors in [19,20] performed an *in vitro* investigation of their electromagnetic sensor; they performed their experiments on animal blood, but not on solutions lacking cellular components. Thus, it is difficult to conclude whether their sensor was able to detect the conductivity variations directly determined by glucose in a solution, and hence a direct comparison with our results is not possible. In fact, the presence of cellular components exposes a fluid sample to specific dispersion phenomena, that were probably present in the experiments performed in studies [19,20], but not in our experiments. In any case, in both static and dynamic conditions, in the analyzed blood samples they found significant variations in the sensor output voltage in relation to variations in blood glucose concentration, and the relationships were almost linear, in agreement with our results. As regards the comparison between the results in static and dynamic conditions, they found very small but not negligible differences, again in agreement with our results. However, it must be noted that, apart for the different type of experiments, the electromagnetic system proposed in [19,20] was very different from ours, and certainly more complex: for instance, it required two electromagnetic subsystems (the measuring subsystems, and the reference subsystems, each requiring a couple of inductors); furthermore, the inductors were much larger than those used in our sensor, and the whole system appear to have size and complexity not compatible with a possible, future application in an *in vivo* context. Another difference is that in [19,20] the best results were found at relatively high frequencies (some MHz), whereas we worked at lower frequencies. Low frequency systems allow possible development of devices of relatively low complexity in terms of design and manufacturing, and also at reduced cost. On the other hand, it must be acknowledged that in [19,20] a wide frequency range was studied, and the region of higher sensitivity to glucose variations was identified. On the contrary, in our study we only worked at fixed frequency (the optimal frequency for the inductors used in the electromagnetic sensor): future studies may be devoted to the analysis of the system performance at different frequencies.

Beside the electromagnetic sensor proposed in [19,20], for measuring variations in glucose concentration there exist also some further electromagnetic sensors, which were described in some patents (such as [21,22]). These patents describe in full details the sensor and the working principle, but no measurement data are reported to be compared with our results.

Further comparison of our results with previous findings may be obtained considering some studies of the glucose-induced variations in the dielectric properties of solutions performed with the traditional, electrode-based approach. In both the studies [5,6] the impedance of glucose-water solutions increased for increasing glucose concentration values, in agreement with our results. In our previous study [6], some 0.9% sodium chloride samples were also studied, and again we found an increase in the impedance for increasing glucose values in a wide frequency range. Of note, at the frequency values where the impedance variations were more evident, the phase of the impedance was almost zero, thus impedance was reduced to pure resistivity/conductivity. This further confirms that limiting the analysis of the glucose-induced variations to pure conductivity, as it was done in this study, may be an acceptable approach.

## 5. Conclusions

We have presented a novel, low frequency electromagnetic sensor for the estimation of variations in glucose concentration of several solutions. The main relevant aspects of this study are the following: (i) we showed that the variations in the conductivity of solutions due to changes in glucose concentration can be observed, even at very low frequencies (40 kHz in this study); (ii) we obtained these results with an electromagnetic sensor, which does not make use of electrodes in touch with the solution, thus avoiding any criticism about electrode polarization phenomena possibly affecting the results; (iii) the specific electromagnetic sensor that we developed is simple and cheap.

Obviously, several further studies, with specific deeper analysis of the glucose concentration range below 400 mg/dL, need to be carried out to elucidate whether this approach may be adequate for possible *in vivo* applications on humans, where several confounding factors may be present and need to be properly accounted for. Furthermore, also the shape of the sensor, in its actual configuration based on the presence of a fluid ring, may result in a drawback for *in vivo* applications. However, on the other hand the sensor appeared to be able to detect the glucose variations in all the solutions analyzed, even at glucose concentration values typical of normoglycaemia in humans.
